# Digital Asthma Self-Management Interventions: A Systematic Review

**DOI:** 10.2196/jmir.2814

**Published:** 2014-02-18

**Authors:** Deborah Morrison, Sally Wyke, Karolina Agur, Euan J Cameron, Robert I Docking, Alison M MacKenzie, Alex McConnachie, Vandana Raghuvir, Neil C Thomson, Frances S Mair

**Affiliations:** ^1^General Practice & Primary CareInstitute of Health & Wellbeing, College of Medical, Veterinary & Life SciencesUniversity of GlasgowGlasgowUnited Kingdom; ^2^Institute of Health & WellbeingCollege of Social SciencesUniversity of GlasgowGlasgowUnited Kingdom; ^3^Respiratory MedicineInstitute of Infection, Immunity & InflammationUniversity of GlasgowGlasgowUnited Kingdom; ^4^Forth Valley Royal HospitalNHS Forth ValleyUnited Kingdom; ^5^Robertson Centre for BiostatisticsInstitute of Health & Wellbeing, College of Medical, Veterinary & Life SciencesUniversity of GlasgowGlasgowUnited Kingdom; ^6^School of MedicineUniversity of GlasgowGlasgowUnited Kingdom

**Keywords:** asthma, self-management, Internet, eHealth, systematic review, patient education

## Abstract

**Background:**

Many people with asthma tolerate symptoms and lifestyle limitations unnecessarily by not utilizing proven therapies. Better support for self-management is known to improve asthma control, and increasingly the Internet and other digital media are being used to deliver that support.

**Objective:**

Our goal was to summarize current knowledge, evidenced through existing systematic reviews, of the effectiveness and implementation of digital self-management support for adults and children with asthma and to examine what features help or hinder the use of these programs.

**Methods:**

A comprehensive search strategy combined 3 facets of search terms: (1) online technology, (2) asthma, and (3) self-management/behavior change/patient experience. We undertook searches of 14 databases, and reference and citation searching. We included qualitative and quantitative systematic reviews about online or computerized interventions facilitating self-management. Title, abstract, full paper screening, and quality appraisal were performed by two researchers independently. Data extraction was undertaken using standardized forms.

**Results:**

A total of 3810 unique papers were identified. Twenty-nine systematic reviews met inclusion criteria: the majority were from the United States (n=12), the rest from United Kingdom (n=6), Canada (n=3), Portugal (n=2), and Australia, France, Spain, Norway, Taiwan, and Greece (1 each). Only 10 systematic reviews fulfilled pre-determined quality standards, describing 19 clinical trials. Interventions were heterogeneous: duration of interventions ranging from single use, to 24-hour access for 12 months, and incorporating varying degrees of health professional involvement. Dropout rates ranged from 5-23%. Four RCTs were aimed at adults (overall range 3-65 years). Participants were inadequately described: socioeconomic status 0/19, ethnicity 6/19, and gender 15/19. No qualitative systematic reviews were included. Meta-analysis was not attempted due to heterogeneity and inadequate information provision within reviews. There was no evidence of harm from digital interventions. All RCTs that examined knowledge (n=2) and activity limitation (n=2) showed improvement in the intervention group. Digital interventions improved markers of self care (5/6), quality of life (4/7), and medication use (2/3). Effects on symptoms (6/12) and school absences (2/4) were equivocal, with no evidence of overall benefits on lung function (2/6), or health service use (2/15). No specific data on economic analyses were provided. Intervention descriptions were generally brief making it impossible to identify which specific “ingredients” of interventions contribute most to improving outcomes.

**Conclusions:**

Digital self-management interventions show promise, with evidence of beneficial effects on some outcomes. There is no evidence about utility in those over 65 years and no information about socioeconomic status of participants, making understanding the “reach” of such interventions difficult. Digital interventions are poorly described within reviews, with insufficient information about barriers and facilitators to their uptake and utilization. To address these gaps, a detailed quantitative systematic review of digital asthma interventions and an examination of the primary qualitative literature are warranted, as well as greater emphasis on economic analysis within trials.

##  Introduction

Asthma is common, affecting an estimated 300 million people worldwide. The number of disability-adjusted life years lost is estimated at 15 million per year, similar to that for diabetes [1]. The main goals of treatment for asthma include achieving and maintaining control of symptoms, normal activity levels, minimal exacerbations, normal lung function, and preventing deaths from asthma [2]. However, these goals are not widely achieved; people with asthma often tolerate unnecessary symptoms, and management of the condition can often be suboptimal [1,3,4]. Guided self-management for asthma as part of systematic, planned care can lead to improvements in patient outcomes such as increases in knowledge, confidence to manage asthma, and improved quality of life, as well as reductions in hospitalizations, emergency room visits, unscheduled visits to the doctor, and days off work or school [5-8].

Despite evidence of benefits, guided self-management, particularly through the use of asthma plans, remains underused [9-11]. While interventions can often be successful in trial settings, evidence of their implementation into every day practice is limited [9,12]. Therefore, there is growing interest in the potential of the Internet and other digital media as a medium to deliver more tailored, relevant self-management support, while maintaining cost-effectiveness, with greater scope for integration into the everyday lives of those with asthma.

While many reviews have been published in the field of self-management in asthma, there is a lack of clarity about the role of digital interventions and which specific components of interventions or “ingredients” contribute most to promoting effective self-management practices and translate into improvement in patient outcomes. There is increasing interest in standardizing methods of determining the “active ingredients” of self-management interventions, potentially making it easier to measure and reproduce those features found to be most effective in future interventions [13].

The aim of this paper is to summarize current knowledge, evidenced through existing systematic reviews, of the effectiveness and implementation of digital self-management support for adults and children with asthma and to examine what features help or hinder the use of these programs. We describe our metareview, which examines the effects, if any, of asthma digital self-management interventions on a range of measures of lung function, symptoms, quality of life, and health care utilization.

##  Methods

### Overview

We conducted a systematic review of systematic reviews—an approach that has proven helpful in synthesizing a broad base of literature in order to identify research gaps and inform future intervention programs [14]. Our aims were to assess the evidence of effectiveness of asthma digital interventions for self-management as measured by an inclusive range of clinical and process outcomes.

We documented recruitment and retention rates, information about implementation processes, whether cost effectiveness was assessed, and whether theories of behavior change were used in intervention development to help us gain a better of understanding of features that helped or hindered the use of the programs. Our protocol is available in Multimedia Appendix 1.

### Inclusion and Exclusion Criteria

We included qualitative and quantitative reviews and used the PICOS (participants, interventions, comparison, outcomes, study design) framework [15] to define inclusion criteria (see Textbox 1 for details). Our exclusion criteria can be found in Textbox 2.

**Inclusion criteria.**

- Participants: those with asthma of any age or their caregivers.
- Intervention: online or computerized interventions facilitating self-management through education and/or providing advice or other behavior change approach. We included only interventions that provided these features independent of any health professional input. Interventions delivered by computer, tablet, smartphone, or purpose-built electronic device were included.
- Comparison: usual care or other forms of self-management interventions, such as face-to-face education or written information.
- Primary outcomes:
  - activity limitation (eg, days off work/school/disturbed nights)
  - adverse events
  - barriers and facilitators to online asthma intervention use by patients and practitioners
  - biomarkers of airway inflammation (eg, exhaled nitric oxide)
  - health service utilization (including scheduled/unscheduled, and primary/secondary care)
  - lung function (eg, spirometry & reversibility, peak expiratory flow [PEF])
  - medication use (eg, relief inhaled β agonist use, compliance with medication)
  - quality of life
  - symptoms (measures of asthma control, eg, diary card scores, asthma control questionnaire, exacerbation rates)
- Secondary outcomes:
  - markers of self-management (eg, adherence to monitoring tools, use of action plans, self-efficacy)
  - patient knowledge
  - patient satisfaction
  - recruitment, retention rates
  - cost effectiveness
  - use of behavior change theory during intervention development and implementation processes
- Study design: Quantitative reviews describing randomized controlled trials (RCTs) and qualitative reviews seeking to understand the patients or providers’ experience of using these asthma interventions and those that describe the theory behind the development of such interventions. The full definition of a systematic review used is found in Multimedia Appendix 1.

**Exclusion criteria.**

- Intervention: interventions consisting only of telemonitoring or clinical decision support software for health professionals were excluded. Interventions that provided only a means of self-monitoring without direct feedback were excluded (eg, electronic diaries for recording peak flows or symptoms that did not provide automated feedback). The content of the intervention was required to be delivered at least in part by the digital medium itself. Devices that were simply digital modes of communicating between patients and health professionals were excluded.
- Outcomes: reviews that did not provide information specific to our outcomes of interest.
- Study design: conference proceedings and theses, and for quantitative reviews, non-RCTs were excluded.

### Information Sources and Search Strategy

A professional systematic review company (York Health Economic Consortium) searched a wide range of databases covering health, mental health, education, social science (14 in total), with no start date before July 2011. The search strategy was devised using a combination of subject indexing terms (eg, MeSH [medical subject headings] in MEDLINE), and free-text search terms in the title and abstract. The search terms were identified through discussion between the research team, by scanning background literature, and by browsing a database’s thesaurus. To ensure sensitivity, the search strategy did not include a methodological search filter to identify reviews. The searches were not limited by date range or language. Hand-searching of Patient Education and Counseling, and the Primary Care Respiratory Journal, and reference searching and citation searching of included studies was undertaken (DM). We contacted experts to establish if any reviews had been missed. The search of electronic databases was updated to October 3, 2013.

The search strategy covered 3 broad areas: (1) asthma and related terms, (2) online/computerized and related terms, and (3) self-care/self-management, patient experience, qualitative, and related terms. The full list of databases searched and an example of the full search strategy for MEDLINE are available in Multimedia Appendix 2.

### Study Selection

Titles, abstracts, and full papers were screened by one researcher (DM) plus one other independent researcher (EC, SW, FM, NCT, KA, RD, AM, or VR). We independently undertook quality appraisal; disagreements were resolved by discussion with a third party if necessary. Only studies meeting predetermined quality criteria advanced to data extraction.

### Data Collection

We used online data collection forms using Distiller SR software. For each included review, we collected (1) general information about the review (year, country of first author, language, number of studies, number of asthma studies, and number of digital asthma studies), (2) descriptions of each included RCT and intervention that fulfilled our criteria (inclusion criteria were applied to the systematic reviews initially and then subsequently to their featured RCTs, to determine those relevant to this review), and (3) results for each outcome of interest and the original article this result was derived from (including quotes from qualitative/narrative reviews).

### Quality Appraisal

Quality appraisal was undertaken in two ways. First, at the full paper screening stage, papers were required to meet criteria laid out in our definition of a review (eg, evidence of a systematic search or criteria for selection of papers must be included; see Multimedia Appendix 1 for full definition). Then, the included papers underwent formal quality appraisal using A Measurement Tool to Assess Systematic Reviews (AMSTAR) [16-18]. This 11-point checklist covers the following domains: establishing the research question and inclusion criteria before the conduct of the review, data extraction by at least 2 independent data extractors, comprehensive literature review with searching of at least two databases, detailed list of included/excluded studies, quality assessment of included studies and consideration of quality assessments in analysis and conclusions, appropriate assessment of homogeneity, assessment of publication bias, and a statement of any conflict of interest. We made minor alterations to the wording in order to make the checklist applicable to non-quantitative reviews (available on request from the corresponding author). Papers needed to achieve at least 50% of quality indicators, plus a “yes” to question 7, which asks “Was the scientific quality of the included studies assessed and documented?” This was required to allow us to make a comment on the quality of the included data. Two researchers (DM + [KA, RD, AM, or VR]) scored each paper independently, with conflicts resolved by discussion with a third party if necessary (FM).

Any data provided describing risk of bias across reviews, and within individual RCTs, were extracted to inform the discussion.

### Data Synthesis

Meta-analysis was not possible. Quantitative results for individual outcomes results were described as favoring the intervention group, favoring the control group, or demonstrating no difference. All results were included in a narrative summary.

##  Results

### Results of Article Screening and Selection

**Figure 1:**
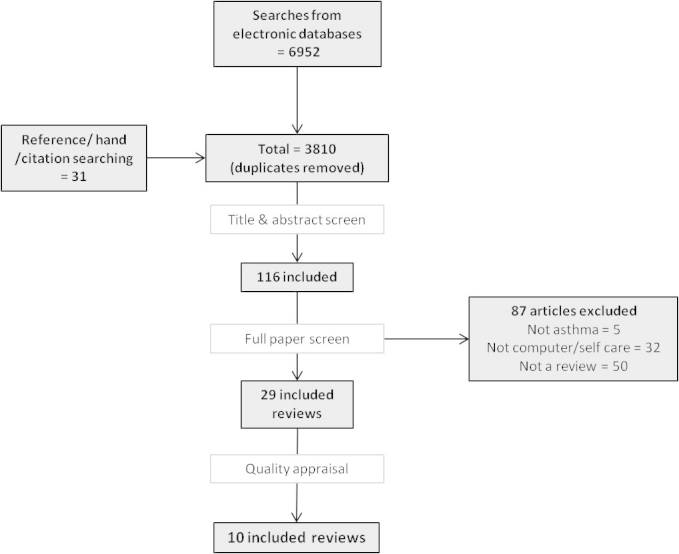
Flow chart demonstrating the screening process of papers in the systematic review.

Our search identified 3810 unique citations, and title and abstract screening identified 116 full papers for review. Of these, 29 fulfilled our inclusion criteria (Figure 1).

### Description of Included Reviews

The 29 papers included systematic reviews from United States (12), United Kingdom (6), Canada (3), Portugal (2) Australia, France, Spain, Norway, Taiwan, and Greece (1 each). Only 9 reviews focused completely on asthma interventions (the others included self-management interventions for asthma alongside a range of other chronic conditions such as diabetes, heart failure, and hypertension), and 11 reviews looked exclusively at digital interventions (the rest examined digital interventions alongside other modes of self-management). These 29 reviews consisted of 4 Cochrane reviews [19-22] and 3 meta-analyses [23-25], with the remaining 22 being narrative or descriptive studies (see Table 1; [10,19-47]).

**Table 1:** Included reviews with quality appraisal results.

First author of systematic review	Year	RCTs, n	Asthma RCTs, n	Digital asthma RCTs, n	Country, language	AMSTAR, %	Question 7=Yes^a^
Krishna et al [26]	1997	22	2	2	United States, English	10	N
Lewis [27]	2003	32	7	4	United States, English	40	N
Wantland et al [23]	2004	20	2	2	United States, English	36	N
Murray et al [19]^b^	2005	24	6	5	United Kingdom, English	82	Y
Almeida et al [28]	2006	13	13	12	Portugal, English	11	N
Bussey-Smith & Rossen [29]	2007	9	9	9	United States, English	30	N
Garcia-Lizana & Sarria-Santamera [30]	2007	24	5	5	Spain, English	40	N
Ring et al [10]^b^	2007	14	14	1	United Kingdom, English	70	Y
Coffman [25]	2008	37	37	6	United States, English	20	N
Fox [31]	2009	25	4	4	United States, English	10	N
Moeinedin et al [32]	2009	27	8	7	Canada, English	40	Y
Papastergiou [33]	2009	34	3	3	Greek, English	20	N
Stinson et al [34]^b^	2009	9	5	3	Canada, English	70	Y
Boyd et al [20]^b^	2009	30	30	2	Australia, English	90	Y
Coscrato et al [35]	2010	16	3	3	Portugal, Portuguese	30	Y
Cushing & Steele [24]	2010	33	9	9	United States, English	45	N
Gremeaux et al [36]	2010	39	4	4	France, English	11	N
McDermott & While [37]^b^	2010	15	2	2	United Kingdom, English	50	Y
McLean et al [22]^b^	2010	21	21	5	United Kingdom, English	100	Y
Pare et al [38]	2010	62	8	1	Canada, English	40	Y
Klasnja et al [39]	2011	n/a^c^	n/a	n/a	United States, English	10	N
Welsh et al [21]^b^	2011	12	12	1	United Kingdom, English	90	Y
Chia-Chi Kuo & Hsiu-Hung Wang [40]	2012	12	4	1	Taiwan, Chinese	30	Y
Chrisler [41]	2012	18	18	6	United States, English	20	N
Johansen et al, (2 part review) [42,43]^b^	2012	29	7	5	Norway, English	60	Y
Kirk et al [44]^b^	2012	13	10	2	United Kingdom, English	50	Y
Mosnaim et al [45]	2012	17	17	10	United States, English	20	N
Nickels & Dimov [46]	2012	3	2	2	United States, English	10	N
Hieftje et al [47]	2013	19	6	6	United States, English	50	Y

^a^AMSTAR Q7 relates to whether the review assesses and documents any quality appraisal of their included studies.

^b^These studies meet appraisal criteria.

^c^Klasnja et al was a qualitative paper and did not specifically provide details of included papers.

### Quality of the Included Reviews

Within the 29 included reviews, descriptions of the included RCTs and particularly the interventions themselves were generally suboptimal. AMSTAR scores ranged from 10% to 100% with a median of 40% (interquartile range 20–50), with 10 reviews scoring 50% or greater. All reviews with 50% or over also provided an individual assessment of quality, which was an essential criterion to progress to data extraction. Areas where reviews performed particularly poorly include providing information about conflict of interests of included RCTs (1/29), providing a priori design (6/29), independent screening and data extraction (6/29 clearly demonstrated this; a further 15/29 may have, but insufficient information was provided to be able to confirm this), and providing a list of excluded studies (7/29).

Of the 10 reviews meeting the predetermined AMSTAR requirements (Table 1), these included the 4 Cochrane reviews and 6 narrative/descriptive reviews. Four reviews focused only on asthma, with the remaining 6 featuring a range of conditions. Four of the reviews included nondigital/electronic modes of delivery of interventions. There was no one review that looked specifically at digital interactive interventions aimed only at those with asthma. These 10 systematic reviews presented results from a total of 19 RCTs meeting our inclusion criteria (see Table 2 for descriptions [10,19-22,34,37,42-44,47-68] and Table 3 for interventions [48-66]).

All 10 systematic reviews provided sample sizes for their included RCTs, but only three provided numbers allocated to intervention group versus control [19,20,37]. All reviews presented some information about age, 8 out of 10 presented information about gender, and only 4 of the 10 reviews about the ethnicity of participants [19-21,44]. No review provided information about socioeconomic status or levels of educational attainment.

**Table 2:** Description of included RCTs within included systematic reviews.

Systematic review	Author (year of RCT)	Sample size, n	Age, years	% male^a^	Ethnicity^b^	Nos. at follow-up	Drop-out rate, %	Duration	Country
McLean 2010 [22]	Cruz-Correia 2007 [48]	21	16-65	n/a	n/a	n/a	n/a	4 wks intervention, 4 wks control	Portugal
	Guendelman 2002 [49]	134	8-16	n/a	n/a	122/134	9	12 wks	United States
	Jan 2007 [50]	196	6-12	n/a	n/a	153/196	22	12 wks	Taiwan
	Rasmussen 2005 [51]	300	18-45	n/a	n/a	253/300	16	52 wks	Denmark
	Van der Meer 2009 [52]	200	18-50	n/a	n/a	183/200	8	52 wks	Netherlands
Stinson 2009 [34]	Jan 2007 [50]	n/a^c^	6-12	38	n/a	n/a	17	12 wks	Taiwan
	Joseph 2007 [53]	n/a^c^	5-19	37	n/a	n/a	17	26 wks	United States
	Krishna 2003 [54]	n/a^c^	7-17	65	n/a	n/a	10	52 wks	United States
Ring 2007 [10]	Rasmussen 2005 [51]	300	18-45	32 34^d^	n/a	253/300	16	n/a	Denmark
Murray 2005^e^[19]	Bartholomew 2000 [55]	171	7-17	65	Hispanic 42%, AA 53%	133/171	22	4 to 15.6 months, mean 7.6 months	United States
	Guendelman 2002 [49]	134	8-16	57	AA 76%	120/134	10	12 wks	United States
	Homer 2000 [56]	137	3-12	69	AA 60.5%, Hispanic 5.3%	106/137	23	Used over 3 sessions at community clinic	United States
	Krishna 2003 [54]	246	7-17	65	White 86%	228/246	7	Approx. 80 minutes to complete. Used during 3 routine clinic visits.	United States
	Shegog 2001 [57]	76	10.7 (mean)	61	White 48%, AA 41%, Hispanic 7%	71/76	7	Game played during session a medical centre	United States
Hieftje 2013 [47]	Bartholomew 2000 [55]	133	7-17	65	n/a	n/a	n/a	15.6 months	n/a
	HussB 2003^f^[58]	148	7-12	44	n/a	n/a	n/a	12 wks	n/a
	McPherson 2006 [59]	101	7-14	53	n/a	n/a	n/a	6 months	n/a
	Rubin 1986 [60]	65	7-12	n/a	n/a	n/a	n/a	10 months	n/a
	Shames 2004 [61]	119	5-12	60	n/a	n/a	n/a	12 months	n/a
	Vilozni 2001 [62]	112	3-6	44	n/a	n/a	n/a	One-off session	n/a
Boyd 2010 [20]	Homer 2000 [56]	137	3-12	69	AA 61%	106/137	23	12 wks (used game 3 times)	United States
	Shames 2004 [61]	119	5-12	58	Hispanic 57%, AA 21%	97/119	18	32 wks	United States
Johansen 2012 [42,43]	Chan 2007 [63]	120	6-17	63	n/a	n/a	n/a	6 wks (follow-up 12 months)	United States
	Guendelman 2002 [49]	134	8-16	40 (i) 37 (c)	n/a	n/a	n/a	Follow-up 3 months	United States
	Jan 2007 [50]	164	6-12	40 (i) 37 (c)	n/a	n/a	n/a	Follow-up 3 months	Taiwan
	Rasmussen 2005 [51]	300	18-45	31	n/a	n/a	n/a	Follow up 6 months	Denmark
	Van der Meer 2009 [52]	200	18-50	31	n/a	n/a	n/a	Follow-up 12 months	Netherlands
Welsh 2011 [21]	Kamps 2004^g^[64]	20	7-12	57 (i) 75 (c)	AA 20%, European American 53%, Hispanic American 27%	15	25	6 wks	United States
McDermott 2013 [37]	Sundberg 2005 [65]	97	18-25	n/a	n/a	n/a	n/a	One 1 hour intervention, follow-up at 1 year	Sweden
	HussA 1992^h^[66]	52	n/a	n/a	n/a	n/a	n/a	1x 22 min session 12 wks	United States
Kirk 2013 [44]	Guendelman 2002 [49]	134	8-16	58	AA 76.1%	128/134	4.5	12 wks	United States
	Jan 2007 [50]	179	6-12	38	n/a	164/179	8.4	12 wks	Taiwan

^a^(i) intervention group; (c) control group

^b^AA=African American.

^c^Stinson provided “participants” and “drop out %” but it is unclear if participants refers to original sample size, or those available for follow-up. Numbers provided were Jan 2007 (164), Joseph 2007 (314), and Krishna 2003 (228).

^d^% provided for individual groups (3-arm trial), however, it stated being unable to provide % for third group due to reporting discrepancies.

^e^Huss 2003: no results were provided in the review for data extraction therefore it was excluded.

^f^There were two trials led by Huss: for the purposes of this review they are referred to as HussA and HussB.

^g^Throughout the Welsh review, this RCT is referred to as “Kamps 2008”, but the reference states 2004.

^h^This review references 3 papers for this trial [66-68], but for the purposes of this review we will reference the most recent publication [66].

**Table 3:** Descriptions of interventions included within reviews (the checkmark indicates evidence of the presence of an intervention component).

First author of RCT, year	Asthma information, self-care education	Asthma action plan	Self-monitoring, eg, PEF, symptoms with things like diaries	Interactive/ immediate feedback from device	Messages /alerts to patients from device	Message/ alert to/from health professionals	Games, quizzes, vignettes	Daily use	Mode of delivery
Rubin 1986 [60]							√		Computer game
HussA 1992 [66]	√			√					Computer program
Bartholomew 2000 [55]	√		√	√			√	√	CD-ROM
Homer 2000 [56]	√		√	√			√	√	Computer game
Shegog 2001 [57]	√		√	√			√	√	CD-ROM
Vilozni 2001 [62]	√						√		Computer game
Guendelman 2002 [49]	√		√	√		√	√	√	Internet enabled device
HussB 2003 [58]	√			√			√		Computer game
Krishna 2003 [54]	√		√	√		√	√	√	Internet enabled CD ROM
Kamps 2004 [64]	√								Computer program
Shames 2004 [61]	√			√			√		Computer game
Sundberg 2005 [65]	√								Computer program
Rasmussen 2005 [51]		√	√	√	√	√			Web-based
McPherson 2006 [59]				√			√		Computer game
Chan 2007 [63]	√					√			Web-based
Cruz-Correia 2007 [48]	√	√	√	√	√	√			Web-based
Jan 2007 [50]	√	√	√	√	√	√			Web-based
Joseph 2007 [53]	√					√			Web-based
Van der Meer 2009 [52]	√	√	√	√	√	√		√	Web-based

### Quality of Evidence in Included Reviews

Seven of the reviews provided risk of bias data based on the guidance provided by the Cochrane collaboration [69], whereby different elements such as adequate sequence generation, allocation concealment, and blinding are assessed as being of low risk of bias, high risk of bias, or unclear risk of bias. This does not provide an overall score. Two reviews provided quality scores, one based on the Oxford Quality scoring system [47] and the other using the Consort Statement [34]. The final review [10] provided an overall quality grade based on guidance from within the Cochrane handbook, but did not provide any rationale for their grading. Grading of RCTs in this way is subjective, and there was conflict between reviews about the risk of bias present in a given RCT. For example, the presence of adequate sequence generation by Guendelman was assessed by 4 different reviews [19,22,42-44]. Two described this as “unclear risk of bias”, but the other two reported it as “adequate”, and “low risk of bias”. This, combined with the various different methods used and in some cases limited information provided, meant that we are unable to make any detailed statements about the quality of the included trials.

### Descriptions of Included Randomized Controlled Trials

The 19 unique RCTs within the reviews are described in Table 2. Reporting of descriptive data about RCT participants was mixed; only 4/10 reviews [19-21,44] provided information for all 7 descriptive headings (sample size, age of participants, gender, ethnicity, dropout rate, duration, country of study), with no data about socioeconomic status. The sample sizes for the 18 RCTs providing quantitative results ranged from 20 to 378. There were discrepancies in the sample sizes reported for some RCTs between reviews; for example, the RCT by Jan had 3 different sample sizes reported from 4 reviews (164, 179, and 196). Where there were discrepancies, taking the largest number provided the total number of participants of 2315. From the 19 RCTs featured, 4 were aimed at adults, 14 at children or adolescents, and the age range for one was not described (HussA). All 4 of the RCTs aimed at adults had upper age limits (of 25, 45, 50, and 65 years).

From the 8 RCTs with information about gender, 9 had a majority of female participants and 9 had a majority of males; combining the RCTs with sample size and gender numbers, the percentage of male participants was 54% (793/1478). Dropout rates were available for 11 of the 19 RCTs and ranged from 4.5% to 23%, but again, the reporting of these numbers for 3 of these RCTs was conflicting (Krishna, Jan, Guendelman). Reasons for dropout were rarely provided. Duration of studies ranged from a one-off use of the intervention, to access for 12 months. Eleven RCTs were from the United States, and one each from Taiwan, Portugal, Sweden, Denmark, and the Netherlands. The country was not described for the remaining 3 RCTs.

### Descriptions of Contents of Included Interventions

A summary of the key components or ingredients reported as being present in the interventions is summarized in Table 3. Provision of information and self-management education was the most common feature present in 16/19 RCTs. This was followed by the presence of immediate feedback/interactivity from the device (13/19).

Ten of the interventions used games/quizzes/vignettes, and all of these were from RCTs from 2006 or earlier. Eight of the interventions involved some form of direct communication either to or from health professionals. Six of the interventions were available for daily use. The presence of an action plan was noted in 4 interventions, all 2005 or later. This was the same for interventions featuring automated reminders/alerts from devices. Description of the interventions was variable between reviews, and it is possible that many of these interventions feature components not described in Table 3.

The key ingredients or components of interventions were often poorly described rendering it impossible to draw conclusions about the effects of different components of interventions on outcomes.

### Results Relating to Outcomes of Interest

There were quantitative results available for the following outcomes of interest: activity limitation, knowledge, markers of self-care, quality of life, medication use, symptoms, missing school, lung function, and health service utilization (eg, emergency department visits, hospitalizations, primary care visits).

There were descriptive results relating to adverse events, behavior change theory use, and patient satisfaction. No data were provided about cost-effectiveness or biomarkers of airway inflammation.

### Results From Quantitative Reviews

Quantitative results were provided in a range of ways across the reviews. For example, McLean et al [22] provided original numbers of events and sample size, with results given as odds ratios with confidence intervals, whereas Stinson et al [34] reported the outcome as either statistically significantly in favor of the intervention group (+), the control group (-), or no difference (0). Murray et al [19] presented results as either standardized mean differences (SMD) and effect size (in the form of Lipsey Categories of small, medium, or large) or odds ratios. However, the significance for individual RCT results was not provided, limiting the conclusions we can draw from this individual review (therefore not included in the text below). Quantitative results are presented in Table 4 and Multimedia Appendix 3 and summarized below. Multimedia Appendix 3 details all the data extracted from the reviews, along with a summary stating whether the results favored the intervention group (Y), showed no difference (0), or statistical significance of results was not provided (n/a). No results favored the control groups. Table 4 summarizes only the results that provide a measure of statistical significance [10,20-22,34,37,42-44,47].

**Table 4:** Table showing statistically significant results (each bullet represents an individual RCT that demonstrates a statistically significant result for a given outcome).

Outcome	Systematic review	RCTs with results showing no difference (sample size)^a,b^	RCTs with results favoring intervention (sample size)^a,b^	Number of RCTs described with a statistically significant result that:		
				Favors control	Shows no difference	Favors intervention
Activity limitation	Johansen [42,43]; Kirk [44]; Stinson [34]		Joseph (314); Guendelman (134)^c^			••
Knowledge	Johansen [42,43]; Kirk [44]; Stinson [34]		Jan (196)^c^; Krishna (228)			••
Markers of self-care	Hieftje [47]; Johansen [42,43]; Kirk [44]; McDermott [37]; Ring [10]; Stinson [34]	Guendelman (134)	Jan (196)^c^; Joseph (314); Rasmussen (300); Rubin (65); Vilonzi (112); HussA (52)		•	••••••
Quality of life	Hieftje [47]; Johansen [42,43],[44]; McLean [22]; Stinson [34]; Welsh [21]	Joseph (314); Krishna (228); Kamps (20)	Jan (196)^c^; Van der Meer (200)^c^; Shames (119); Rasmussen (300)		•••	••••
Medication use	Hieftje [47]; McDermott [37]	Shames (119)	McPherson (101); HussA (52)		•	••
Symptoms	Hieftje [47]; Johansen [42,43]; Kirk [44]; McDermott [37]; McLean [22]; Stinson [34]; Welsh [21]	Guendelman (134); HussA (52); Shames (119); Kamps (20); Sundberg (97); HussB (148)	Jan (196)^c^; Joseph (314); Krishna (228); Rasmussen (300)^c^; Van der Meer (200)^c^; Bartholomew (171)		••••••	••••••
Missing school	Hieftje [47]; McLean [22]; Stinson [34]	Guendelman (134); Rubin (65)	Joseph (314); McPherson(101)		••	••
Lung function	Boyd [20]; Johansen [42,43]; Kirk [44]; McDermott [37]; McLean [22]; Welsh [21]	Jan (196)^c^; Shames (119); Kamps (20); Huss (20)	Rasmussen (300)^c^; Guendelman (134)^c^; Sundberg (97)		••••	•••
Emergency department visits	Hieftje [47]; Johansen [42,43]; Kirk [44]; McLean [22]; Stinson [34]	Rasmussen (300); Bartholomew (171); Jan (196); Guendelman (134)^c^	Joseph (314); Krishna (228)		••••	••
Hospitalization	Hieftje [47]; Johansen [42,43]; Kirk [44]; McLean [22]; Stinson [34]	Guendelman (134)^c^; Joseph (314); Rasmussen (300); Rubin (65); McPherson (101)			•••••	
Primary care visits	Hieftje [47]; Kirk [44]	Shames (119); Rubin (65); McPherson (101); Jan (196)			••••	

^a^References for individual RCTs as per Table 3.

^b^Where there is discrepancy in sample size reporting, the largest sample size is used.

^c^RCT present in more than one systematic review for a given outcome.

### Activity Limitation

Stinson [34], Johansen et al [42,43], and Kirk [44] reported findings from two RCTs (Joseph and Guendelman). Both reported that the use of a digital intervention reduced the number of days of restricted activity significantly.

### Knowledge

Three reviews [34,42-44] provided results from 2 RCTs (Jan, Krishna) about the impact of online interventions on knowledge, both of which provided results in favor of the use of digital interventions.

### Markers for Self-Management

Six of the reviews [10,34,37,42-44,47] presented data for markers of self-management from 7 separate RCTs (Jan, Joseph, Rubin, Vilozni, Guendelman, HussA & Rasmussen) covering, for example, proportion using action plans, spirometry/inhaler technique, diary adherence, and impact on completion of self-management sessions. All except one (Guendelman) showed a positive effect.

### Quality of Life

Six reviews [21,22,34,42-44,47] presented data from 7 RCTs for quality of life. Four of the RCTs favored the intervention group (Jan, Van der Meer, Shames, Rasmussen), and 3 showed no difference (Joseph, Krishna, Kamps).

### Medication Use

Two reviews [37,47] provided results from 3 RCTs for this outcome. Two favored the interventions group (McPherson & Huss), while the other showed no difference (Shames).

### Symptoms and Asthma Control

Seven systematic reviews [21,22,34,37,42-44,47] provided results for this outcome, from 12 RCTs. Six reported no difference (Guendelman, HussA, HussB, Shames, Kamps, Sundberg); the remaining 6 studies favored the use of the digital interventions (Jan, Joseph, Krishna, Rasmussen, Van der Meer, Bartholomew).

### Missing School

Three reviews reported for this outcome [22,34,47] from 4 RCTS: two favoring the intervention group (Joseph & McPherson), and two showing no difference (Guendelman & Rubin).

### Lung Function

Six reviews [20-22,37,42-44] presented data for this outcome from 7 RCTs, with three favoring the use of digital interventions (Rasmussen, Guendelman, Sundberg), and 4 showing no difference (Jan, Shames, Kamps, HussA).

### Emergency Department Visits

Five reviews [22,34,42-44,47] provided results from 6 RCTs on emergency department (ED) visits. Four trials showed no difference (Rasmussen, Bartholomew, Jan, Guendelman), and 2 studies favored the intervention (Joseph, Krishna).

### Hospitalization

Five reviews [22,34,42-44,47] provided information from 5 RCTs, all of which showed no significant differences between the intervention group and controls (Guendelman, Joseph, Rubin, McPherson, Rasmussen).

### Primary Care Visits

Two reviews provided data for this outcome [44,47] from 4 RCTs (Shames, Rubin, McPherson & Jan). All 4 RCTs demonstrated no difference in the number of visits.

### Outcomes With Descriptive Results Only

#### Adverse Events

Only two reviews [22,37] provided results for this outcome. McLean et al [22] results were specific to one study (Rasmussen) and found that the increased corticosteroid dose/use that went along with being in the intervention or specialist group, compared to the GP group, meant a higher proportion (no details provided) of those patients experienced dysphonia or oral candidiasis. McDermott made comments more generally stating that there appeared to be no adverse outcomes from moving towards computer-based patient self-management programs.

#### Use of Behavior Change Theory During Development

Two systematic reviews explicitly sought to establish the presence or absence of underlying theory in the development of their included interventions [42-44]. Johansen et al [42,43] included as part of their quality appraisal whether or not there was “theoretical evidence that the intervention might have the desired effect”. Of the 5 digital asthma interventions included in their review, all 5 met this criterion (Chan, Guendelman, Jan, Rasmussen, Van der Meer). Kirk et al [44] described the presence or absence of an “underlying theoretical basis” in their included RCTs. They reported that both the digital asthma RCTs in their review (Jan and Guendelman) demonstrated no theoretical basis, which contradicts the findings of Johansen et al [42,43].

Three other reviews described the contents of their included interventions using terminology relevant to behavior change theory. For example, within Murray et al [19], the Shegog trial was described as providing “intensive, tailored information on self-management for children with asthma. Text, graphics, animation, sound and video clips are utilised, and behavior support delivered via verbal reinforcement, guided practice, feedback goal setting and incentives”. However, it does not explicitly describe any role of behavior change theory during development of the intervention.

Stinson et al [34] reported that all three of their included interventions (Jan, Joseph, Krishna) featured training around symptom management, trigger avoidance, and medication use, but that Krishna was the only one described as including behavioral therapy (featuring modeling, reinforcement, and self-mastery), but again no explicit descriptions of theory used during development.

McDermott et al [37] did not describe the presence or absence of behavior change theories during the development of interventions included in their review but did attempt to characterize the “active ingredients” of each included intervention by coding their descriptions using a behavior change technique (BCT) taxonomy. One intervention (HussA) was found to include only one BCT (provide instruction) while the other (Sundberg) was found to have 3 BCTs (provide general information on the condition, advise on medication, and provide instruction).

#### Patient Satisfaction, Barriers, and Facilitators to Digital Asthma Interventions

Three reviews [22,37,42,43] addressed these issues. One study (Cruz-Correia) featured within the McLean et al [22] review found that patients preferred a Web-based system of monitoring asthma compared to a paper-based system. Johansen et al [42,43] reported in general that “an interactive-feedback learning mechanism can provide the stimulus for the patient to build the necessary confidence to handle symptoms and self-management, and in this way support patient centeredness”, and they reported specifically that the Guendelman study suggested the electronic devices might be considered as a “motivating and exciting tool for children with asthma”. Johansen et al [42,43] also reported the findings from the trial by Jan that children found their tool to be “fun” and concluded more research was required in this area. McDermott et al [37] reported findings in general suggesting that computer-based programs may “even be preferred by many patients as they allow participants to proceed at their own pace”. McDermott et al [37] also commented that the combination of standard and computer-based approaches might seem to be the ideal scenario, but state there was no evidence found in their review that is the case.

Only one review mentioned implementability of interventions. Kirk et al [44] felt that the integration of the intervention featured in the trial led by Jan was feasible in current practice but did not elaborate.

#### Cost Effectiveness Analysis

Three Cochrane reviews [19,20,22] and one narrative synthesis planned to include this outcome measure. McLean et al [22] looked at “costs from the health care perspective”, but there were no results specific to interventions matching our inclusion criteria. Murray et al [19] looked at “economic outcomes”, including health care use under this heading. Other than providing summaries of health care use for 3 trials (Bartholomew, Guendelman, Krishna), there was no additional specific cost data provided. Boyd et al [20] planned to collect data on cost, but no studies provided such information. McDermott et al [37] found only one of their included studies provided data on cost, and it was for an intervention aimed at those with diabetes.

## Discussion

### Principal Findings

Initial full paper screening resulted in 29 systematic reviews. However, following quality appraisal, only 10 met the predetermined AMSTAR cut-off values. This metareview summarizes the findings from these 10 systematic reviews featuring a total of 18 unique RCTs of digital asthma self-management interventions. Only 11 of the 19 RCTs were present in more than one review suggesting that at present there is no single good quality systematic review looking specifically at digital self-management interventions for asthma.

Within the systematic reviews we found that information about methods, intervention components, and results were often brief and did not allow for meaningful comparisons between interventions. The described studies themselves were extremely heterogeneous making comparison between interventions difficult. “Control” groups ranged from no active intervention or contact with health professionals to multiple face-to-face teaching sessions and intermittent use of the intervention itself, which may have masked potential positive outcomes of digital interventions. More recent reviews tended to have more information, particularly with online appendices.

In no studies did the control groups have better outcomes, although only two reviews addressed the issue of adverse events, and specific information was available from only one study, which is a concern. That study suggested digital interventions groups may be at higher risk of adverse events related to the fact that successful interventions usually increase inhaled corticosteroid use (a positive outcome) and therefore results in more cases of dysphonia or oral candidiasis. Surprisingly, the issue of patient satisfaction was also neglected, being specifically addressed in only one trial, which suggested participants preferred a Web-based system to a paper-based one, and discussed in general terms in one further review.

Descriptions of intervention development and particularly the use of theory (which has been shown to increase effectiveness) were either brief or not discussed at all, although our review suggests that more recent reviews are increasingly recognizing this, an issue highlighted by the recent publication of a CONSORT EHEALTH statement [70]. This matters because trying to establish the key components of effective self-management interventions is a challenge faced by researchers in this area [71]. For example, a recent Cochrane review of computer-based interventions in adults with type 2 diabetes concluded that there was a small beneficial effect on blood glucose control, particularly in the mobile phone subgroup [72]. However, the authors also commented that the key “ingredient” of effective interventions was unclear; it could either have been that the mobile phone itself was important or that the interventions delivered using mobile phones had included BCTs that were likely to lead to success, and these could be the “ingredient” that made these interventions more effective. What is consistent across several systematic reviews on digital support for self-management of chronic illness is that interventions with multiple behavior change techniques appear, on the whole, to be more effective than those using fewer and that the use of theory to inform the choice and combination of BCTs appears to be associated with increasing effectiveness [72-74].

Only four RCTs featured adults, and there were no participants over the age of 65 years, with only one small trial with just 21 participants including individuals over the age of 50 years. No information was provided about the socioeconomic status or education level of participants. None of the trials were undertaken in low-income countries. Attrition rates of up to 23% were recorded. This is in keeping with other systematic reviews of digital interventions in other areas. For example, a recent Cochrane review examining computer-based weight loss interventions found attrition rates ranging from 2-25% (median 16%) [75], and attrition may be worse in interventions targeting older age groups, with one review including digital and nondigital interventions noting rates between 0 and 52% (median 15%) [73]. Reassuringly, attrition rates are no worse than those found with nondigital self-management asthma interventions as described in Gibson’s Cochrane review examining asthma self-management education and regular health professional review, where attrition rates ranged from 0-54% (median 15%) [8].

Our metareview suggests that digital interventions may be effective at improving knowledge, reducing activity limitation, improving markers of self-management, improving quality of life, and optimizing medication use in those less than 65 years of age. However, certain indicators, such as knowledge and activity limitation, were assessed in only two trials, and medication use improvements noted in two out of three trials that examined this. There was no evidence of improvements in symptoms, lung function, school absences, or health service utilization.

Importantly, we found no qualitative synthesis of asthma digital interventions that would have given insight into the patient experience of using digital self-management interventions, implementation processes, or barriers and facilitators to their use, which is an important gap in the evidence base.

### Strengths and Limitations

Our metareview has a number of strengths and limitations. The search was undertaken by a team with good experience of systematic reviews, using multiple databases, and using a strategy designed iteratively with researchers to be as inclusive as possible, without being unwieldy. Despite this, we may have missed reviews of chronic illness interventions including asthma but not specifically indexed as such. We included non-English studies, which is a strength. Due to the heterogeneity of the data, a formal metasynthesis was not possible.

A further limitation here is the reliability and comprehensibility of the included information. When undertaking a review of reviews, the data are an extra step away from the original research increasing the possibility of reporting errors. We noted several discrepancies between reviews describing the same RCTs, in sample sizes and gender descriptions. Some trials presented data as sample size, while others used terms such as number of participants, and dropout calculations did not appear to correlate on several occasions. This could be due to reporting error, or differences in the interpretation of the terms sample size, follow-up, and participants. In addition, there was a lack of detail describing the populations included, and limited definitions of outcome measures provided by featured systematic reviews. This translates into a lack of specific detail about the population our results are relevant to, and limitations in comparing results that have potentially used different ways of defining outcomes. Finally, establishing a minimum standard of quality of included reviews ensures that there is a degree of reliability to the conclusions [76]; however, this undoubtedly narrowed the available data from which we could draw conclusions. Had we not used AMSTAR criteria, we could have had data from up to a further 12 RCTs and two qualitative studies. However many of these RCTs were included only in low AMSTAR scoring papers (ie, those that had not undertaken any quality appraisal of their included studies), and there were often no descriptions of control groups, or more than a few words describing the interventions, and therefore inclusion of any such data would have rendered our conclusions meaningless. The RCTs themselves often appeared poor quality (eg, poor randomization strategies), with small numbers (eg, sample sizes as low as 10), and had been excluded from the more robust reviews for these reasons. The lack of economic data is a weakness, although the results on health care resource use (hospitalizations and ED visits) suggests that evidence of cost-effectiveness may be lacking. However, without data including routine health care resource utilization and formal economic analysis, no firm conclusions can be drawn.

### Conclusions

This metareview provides a snapshot of current knowledge about effectiveness of digital self-management support for those with asthma. Digital self-management interventions show promise, with evidence of beneficial effects on some outcomes. However, we know nothing about the socioeconomic status of participants, and few over the age of 50 years and no one over 65 years of age were included. Thus, the true “reach” of these studies is uncertain, and their likely uptake and use by the wider population of those with asthma remains uncertain. Few interventions were underpinned by robust theoretical frameworks. Digital interventions are poorly described, and there is insufficient information about barriers and facilitators to their uptake and utilization. Importantly, patient perspectives have been largely ignored in currently available reviews. There was little data about cost effectiveness within reviews, but this appears to relate to the lack of existence of such data from primary trials. Digital interventions for asthma appear promising but further robust investigation is needed, first, in the form of a detailed systematic review of currently available digital interventions aimed at those with asthma, detailing the presence or absence of BCTs. Second, examination of the primary qualitative literature to describe what is already known about the patient’s perspective would be invaluable to inform future interventions.

## Multimedia Appendix 1

Full systematic review protocol.

## Multimedia Appendix 2

Details and results of databases searched, and sample search strategy for Medline Database.

## Multimedia Appendix 3

Description of full quantitative results per outcome.

## Abbreviations

AMSTAR: A Measurement Tool to Assess Systematic Reviews
ACQ: Asthma Control Questionnaire
AQLQ: Asthma Quality of Life Questionnaire
BCT: behavior change techniques
ED: emergency department
FEV1: forced expiratory volume in 1 second
OR: odds ratio
PEF: peak expiratory flow rate
PICOS: participants, interventions, comparison, outcomes, study design
RCT: randomized controlled trial
SMD: standardized mean difference
