# Supplementary material for: Digital Asthma Self-Management Interventions: A Systematic Review
Source: J Med Internet Res. 2014 Feb 18;16(2):e51. doi: 10.2196/jmir.2814 (PMC3958674; doi:10.2196/jmir.2814)
Supplement: Supplementary file 1 [file jmir_v16i2e51_app1.pdf]

Multi Media Appendix 1 – Protocol

Title: A Qualitative and Quantitative Systematic Review of Reviews of the Use of Online/Web-based/Computerised Asthma Self-care Interventions.

Deborah Morrison, Karolina Agur, Neil Thomson, Sally Wyke, Alex McConnachie, Frances Mair

Review question

What is known about the evidence that web-based/online/computerised tools for self management of asthma can improve indices of asthma control, lung function, health care utilisation, patient quality of life, and patient satisfaction, and what helps or hinders the use of such interventions by patients, carers and health professionals.

Objectives

- To undertake a systematic review of all published reviews (quantitative and qualitative) of web-based/online/computerised self-management asthma interventions.
- To establish if the use of web-based/online/computerised self care interventions have been found to have a positive effect on asthma symptom scores, lung function, medication use, health care utilisation, or asthma quality of life scores.
- To identify the presence of techniques in these interventions known to promote behavioural change e.g. educational information, self monitoring, attitudinal arguments, and the use of prompts.
- To examine what factors, if any, have been identified as promoting or inhibiting the uptake and utilisation of online tools by patients, carers and practitioners?

Searches

- Databases to be searched: MEDLINE, EMBASE, CINAHL, PsycINFO, ERIC, Cochrane Library (including CDSR, DARE, Central, and HTA databases), DoPHER and TROPHI (both produced by the EPPI Centre), Social Science Citation Index and Science Citation Index. These databases will be searched using a combination of subject headings where available (such as MeSH) and words in the title and abstracts.

The search strategy combines 3 facets of search terms:

1. Online technology
2. Asthma
3. Self management/behavior change/patient experience

Searches employing more general terms, such as respiratory tract diseases, will be explored as they may identify records where in the full document it becomes clear that patients with asthma are included.

To minimise the risk of missing relevant reviews a manual search of key resources and journals and of the reference lists of reviews captured by initial searches will be undertaken. The search can also be complemented by contacting experts in the topic under review and by carrying out citation searches for articles which cite individual studies that are known to be relevant to the topic.

Types of study to be included/excluded

Included:

Reviews (qualitative and quantitative) describing the use of online/web-based/computerised decision support software interventions providing education and advice on managing asthma for patients with asthma, or their carers. Quantitative reviews which describe RCTs, and qualitative reviews which seek to understand the patients or providers' experience of using these asthma interventions, and those which describe the theory behind the development of such interventions.

Excluded:

- Studies examining clinical decision support software for health professionals.
- Where a review features online/computerised asthma interventions, but the results are indistinguishable from non asthma interventions, or non online/computerised interventions, these papers will be excluded.
- Conference proceedings and theses are excluded.

Condition or domain being studied

Asthma is common, and Scotland has the highest prevalence of asthma symptoms in the world, with patients accepting higher levels of symptoms and lifestyle limitations than they need to, often as a result of not making full use of proven treatment strategies. The promotion of self-care is a strategy known to improve asthma control, and the use of mediums such as the internet and mobile phones are increasingly being considered as a tool to augment its use.

This systematic review of reviews will deliver a position paper on the current knowledge regarding the use of online/web-based/computerised asthma self management tools, and identify gaps in the literature.

Participants/ population

Quantitative and qualitative studies from any geographical location, participants diagnosed with asthma; being treated in any setting: primary; secondary; tertiary care, e.g. in the hospital, community, home; describing a review of online/web-based/computerised asthma interventions.

Intervention(s), exposure(s)

Any review describing the use of online/web-based/computerised asthma interventions to facilitate patients to manage their asthma.

We considered any digital mode of delivery so long as the intervention itself was providing some degree of information or feedback. It needed to be more than telemonitoring, i.e more than a method of communication between users and health professionals. For example a computer programme that collected symptoms or peak flow data to allow a health professional to provide feedback would be excluded.

Comparator(s)/ control

Any comparison with usual care, or alternative modes of delivery of selfmanagement information/skills to participants with asthma or their carers.

Outcome(s)

Primary outcomes may include:

- Measures of asthma control, Symptoms (e.g. diary card scores)
- Measures of asthma quality of life
- Exacerbations
- Restricted activities (e.g. days of work/school/disturbed nights)
- Lung function: e.g. spirometry & reversibility, peak expiratory flow
- Medication utilisation –
- relief inhaled agonist use
- Compliance with medication
- Health service utilisation (including scheduled/unscheduled, and primary/secondary care)
- Biomarkers of airway inflammation (e.g. exhaled nitric oxide)
- Facilitators of online asthma intervention use by patients and practitioners
- Barriers to online asthma intervention use by patients and practitioners
- Adverse events

Secondary outcomes may include

- What behavioural change theories are used, if any, to inform online asthma interventions
- Patient satisfaction
- Patient knowledge
- Adherence to monitoring tools
- Recruitment Retention rates
- Markers of self care (action plan use, inhaler technique for example)
- Data about economic benefits

Study Design - Include review papers only.

Definition of a review

We considered a review paper to be one that provides an analytic account of the research literature related to a specific topic or closely related set of topics. It is intended to contribute to knowledge by answering a research question. Thus we include the following types of papers:

1. Systematic reviews: where relevant literature has been identified by means of structured search of bibliographic and other databases; where transparent methodological criteria are used to exclude papers that do not meet an explicit methodological benchmark, and which presents rigorous conclusions about outcomes.
2. Narrative reviews: where relevant literature has been purposively sampled from a field of research; where theoretical or topical criteria are used to include papers on the grounds of type, relevance, and perceived significance; with the aim of summarising, discussing, and critiquing conclusions.
3. Qualitative metasyntheses or meta-ethnographies, where relevant literature has been identified by means of a structured search of bibliographic and other databases, where transparent methods had been used to draw together theoretical products, with the aim of elaborating and extending theory.

We excluded the following:
1. Secondary analyses (including qualitative metasyntheses or metaethnographies) of existing data-sets for the purposes of presenting cumulative outcomes from personal research programmes.
2. Secondary analyses (including qualitative metasyntheses or metaethnographies) of existing data-sets for the purposes of presenting integrative outcomes from different research programmes.
3. Discussions of literature included in contributions to theory building or critique.
4. Summaries of literature for the purposes of information or commentary.
5. Editorial discussions that argue the case for a field of research or a course of action.

Where the abstract states it is a review, but there is no supporting evidence in the main paper, such as details of databases searched or criteria for selection of papers (either on methodological or theoretical grounds), the paper is excluded.

Data extraction, (selection and coding)

Title, abstract and full paper screening will be carried out by two researchers independently using Distiller software. The full text of the potentially relevant studies will be retrieved and assessed independently for inclusion as per criteria mentioned. Excluded studies will be listed

with reasons of exclusion. Data extraction and data analysis will be carried out using a combination of Distiller software, NVivo software and Microsoft Word. Any disagreements will be resolved by discussion, with a third party if necessary.

Risk of bias (quality) assessment

The AMSTAR tool has been validated as a means to assess the methodological quality of systematic reviews included, and will be utilised during the quality appraisal of included studies [1]. Those achieving 50% plus a 'yes' to question 7 will be included, with appropriate concessions for qualitative studies.

Strategy for data synthesis

Numerical data, e.g. the total number of participants will be analysed using descriptive statistics.

Outcomes from the quantitative reviews will be analysed using appropriate statistical methods. Clinical and methodological heterogeneity will be assessed before pooling.

Findings from the qualitative reviews will be extracted verbatim. A coding frame will be developed to undertake a content analysis of the extracted data from the included reviews.

Analysis of subgroups or subsets

None planned

Dissemination plans

The findings from this work will be disseminated through traditional academic media of conferences and peer reviewed journals but will also be circulated to relevant NHS bodies , charity partners (Asthma UK, British Lung Foundation), and other key bodies such as Quality Improvement Scotland.

Contact details for further information

Deborah Morrison
Academic Unit of General Practice and Primary Care
University of Glasgow

1 Horselethill Road
Glasgow, G12 9LX, UK
Deborah.Morrison@glasgow.ac.uk

Organisational affiliation of the review – University of Glasgow

Review team
Dr Deborah Morrison, University of Glasgow
Euan Cameron, University of Glasgow
Karolina Agur, University of Glasgow
Prof Thomson, University of Glasgow
Prof Wyke, University of Glasgow
Dr Alex McConnachie, University of Glasgow
Professor Frances Mair, University of Glasgow

Other Information:

Details of any existing review of the same topic by the same authors - None

Anticipated or actual start date - August 2011

Anticipated completion date - July 2012

Funding sources/sponsors - Chief Scientist Office, Scotland

Conflicts of interest - None

Other registration details - None

Language - English

Country - Scotland

Key words - Asthma, self care, internet, web-based, online, computerised, quantitative, qualitative, patient education

---

Protocol Amendment October 2013.

Addition of search terms to.

- Keyword searches for text messaging were added;
- MeSH (Medical Subject Headings) terms “Cellular Phone” and “Social Networking” were added;

- The search terms used for mobile phones were enhanced with the addition of “smartphone$ or smart-phone$ or smart-telephone$” and associated terminology such as iPhone, app(s), Apple, Android and Blackberry;
- Newer technologies including tablet devices and social media tools were added as keyword terms;
- The terms “m-health” and “mhealth” were added to search line 29 to reflect the emergence of a new sub-field of e-health concerned specifically with mobile devices.

Electronic search updated to October 2013

Review team updated: R Docking, AM MacKenzie, V Raghuvir joined.

Completion date – November 2013.

Reference List

1. Shea BJ, Hamel C, Wells GA, Bouter LM, Kristjansson E, Grimshaw J, Henry DA, Boers M (2009) AMSTAR is a reliable and valid measurement tool to assess the methodological quality of systematic reviews. Journal of Clinical Epidemiology 62: 1013-1020. doi: 10.1016/j.jclinepi.2008.10.009.
