# Supplementary material for: Digital Asthma Self-Management Interventions: A Systematic Review
Source: J Med Internet Res. 2014 Feb 18;16(2):e51. doi: 10.2196/jmir.2814 (PMC3958674; doi:10.2196/jmir.2814)
Supplement: Supplementary file 2 [file jmir_v16i2e51_app2.pdf]

Multi Media Appendix 2– Example of full search strategy and results.

The following databases and resources were searched:

- MEDLINE and MEDLINE In-Process
- EMBASE
- CINAHL
- PsycINFO
- Cochrane Database of Systematic Reviews (CDSR)
- Database of Abstracts of reviews of Effects (DARE)
- Cochrane Central Register of Controlled Trials (CENTRAL)
- Health Technology Assessment (HTA) database
- NHS Economic Evaluation Database (NHS EED)
- ERIC (Education Resources Information Center)
- Science Citation Index (SCI)
- Social Science Citation Index (SSCI)
- DoPHER
- TRoPHI

Database results

Resource	Number of results 2013
MEDLINE and MEDLINE In-Process	1590
EMBASE	2426
CINAHL	1020
PsycINFO	155
Cochrane Database of Systematic Reviews (CDSR)	15
Database of Abstracts of reviews of Effects (DARE)	2
Cochrane Central Register of Controlled Trials (CENTRAL)	247
Health Technology Assessment (HTA) database	0
NHS Economic Evaluation Database (NHS EED)	4
ERIC (Education Resources Information Center)	16
Science Citation Index (SCI)	1112
Social Science Citation Index (SSCI)	331
DoPHER	12
TRoPHI	22
Total	6952
Total once duplicates removed	3798

Sample search strategy

The search strategy used in MEDLINE (OvidSP) is shown in below (October 2013). This was adapted appropriately to run in the other databases searched.

1 (Computer or computers).hw.
2 exp computers/
3 exp Computer Systems/
4 Medical Informatics/
5 Medical Informatics Applications/
6 Decision Support Techniques/
7 Educational Technology/
8 Audiovisual Aids/
9 Telecommunications/
10 Multimedia/
11 Computer-Assisted Instruction/
12 User-Computer Interface/
13 Hypermedia/
14 Video Games/
15 Electronic Health Records/
16 Cellular Phone/
17 Social Networking/
18 (computer$ or microcomputer$ or PC or PCs or Mac or Macs or Internet or WWW or web or website$1 or webpage$ or local area network$).ti,ab.
19 software.ti,ab.
20 (cellular phone$1 or cellular telephone$1 or mobile$1 or cell phone$1 or cell telephone$1 or smartphone$ or smart-phone$ or smart-telephone$).ti,ab.
21 (handset$ or hand-set$ or wireless or wire-less or wifi or wi-fi or GPS or global positioning system$ or bluetooth or text messag$ or texting or SMS or short messag$ or multimedia messag$ or multi-media messag$ or mms or instant messag$ or social media$ or facebook or twitter or webcast$ or webinar$ or podcast$ or wiki or wikis or app or apps or Android$ or Blackberr$ or Apple$ or iOS or iphone$ or ipad$ or S40 or Symbian$ or Windows).ti,ab.
22 ((electronic$ or digital$ or device$) adj2 tablet$).ti,ab.
23 (video$ or DVD or DVDs).ti,ab.
24 (youtube or you tube or vimeo).ti,ab.
25 (online or on line or interactive).ti,ab.
26 (chat room$1 or chatroom$1).ti,ab.
27 (blog$1 or web-log$1 or weblog$1).ti,ab.
28 (bulletin board$1 or bulletinboard$1 or messageboard$1 or message board$1).ti,ab.
29 (ehealth or e-health or mhealth or m-health).ti,ab.
30 or/1-29
31 exp Asthma/
32 (asthma or asthmatic$1).ti,ab.
33 exp Anti-Asthmatic Agents/ or exp Bronchodilator Agents/
34 or/31-33
35 (action plan or action plans).ti,ab.
36 (self management or self managing).ti,ab.
37 (patient$1 adj3 manag$).ti,ab.
38 health education/
39 education.ti,ab.
40 self care/ or self administration/ or self medication/
41 self care.ti,ab.
42 self monitor$.ti,ab.
43 self treat$.ti,ab.
44 (behavio?r$ adj3 (chang$ or modif$ or condition$)).ti,ab.
45 Patient Satisfaction/
46 (patient$ adj3 (experience$ or attitude$ or view$1 or satisfaction$)).ti,ab.
47 Qualitative research/
48 exp Questionnaires/
49 exp Interviews as Topic/
50 qualitative.ti,ab.

51 (interview$ or questionnaire$ or focus group$).ti,ab.
52 or/35-51
53 30 and 34 and 52
54 animals/ not humans/
55 53 not 54

Key:

/	indicates a subject heading
exp	indicates an exploded subject heading
$	truncation symbol
adj3	words must appear with 3 words of each other
.ti,ab.	searches are restricted to the title and abstract fields
or/1-26	combine sets 1 to 26 using OR
