# Supplementary material for: Digital Asthma Self-Management Interventions: A Systematic Review
Source: J Med Internet Res. 2014 Feb 18;16(2):e51. doi: 10.2196/jmir.2814 (PMC3958674; doi:10.2196/jmir.2814)
Supplement: Supplementary file 3 [file jmir_v16i2e51_app3.pdf]

Multimedia Appendix 3. Description of full quantitative results per outcome.

**OUTCOME: RESTRICTED / LIMITED ACTIVITIES**			
**Review**	**RCT**	**Results**	**Favors intervention group? [a]**
Stinson et al.	Joseph 2007	days of restricted activity/2 weeks: +<br>days of restricted activity : +	Y
Murray et al.	Guendelman 2002	Reduction in limitations in activity.<br>OR: 1.84 at 12 weeks. (OR>1 favours intervention)	n/a
Johansen et al.	Guendelman 2002	After adjusting for covariates, the odds of having any limitation in activity were significantly (P = .03) lower for Health Buddy children. Patients experiencing limitations in activity due to asthma last 14 days: Between-group pre–post change, –9% P = .03.	Y
Kirk et al.	Guendelman	Limitations in activity lower in intervention than control group (OR 0.52; 95% CI 0.29, 0.94; *P* = 0.03).	Y

**OUTCOME: MISSING SCHOOL**			
**Review**	**RCT**	**Results**	**Favors intervention group? [a]**
MacLean et al.	Guendelman 2002	" the odds of missing school in the past six weeks in the Health Buddy group [intervention group] were 0.74 (95%CI 0.37 to 1.5)."	0
Stinson et al.	Joseph 2007	days of school missed/30 days: +	Y
Hieftje et al.	McPherson 2006	At one month no significant difference in school absence between control and intervention groups, but at 6 months lower percent reporting any school absence (OR 2.394, 95% CI 1.021- 5.618)	Y
	Rubin 1986	No change in school absences noted	0

**OUTCOME: PRIMARY CARE VISITS**			
**Review**	**RCT**	**Results**	**Favors intervention group? [a]**
Hieftje et al	Shames 2004	No difference in urgent care visits	0
	Rubin 1986	No difference in visits to the doctor	0
	McPherson 2006	No difference in GP visits at one month	0
Kirk et al	Jan 2007	The effect of the interventions on use of Accident & Emergency Department attendance, and general practitioner/primary care consultations …..four studies reporting no effect. [including Jan 2007]	0

**OUTCOME: HOSPITALISATION**			
**Review**	**RCT**	**Results**	**Favors intervention group? [a]**
MacLean et al	Guendelman 2002	Proportion hospitalization within 3 months of study:<br>Intervention group: events 4, participants 62<br>Control group: events 1, participants 60<br>Odds ratio: 4.07 (0.44 - 37.50) (M-H, Random 95% CI)	0
	Rasmussen 2005	Proportion hospitalisation within 12 months of study:<br>Intervention group: events 0, participants 85<br>Control group: events 1, participants 168<br>Odds ratio: 0.65 (0.03 - 16.20) (M-H, Fixed 95% CI)	0

Stinson et al	Joseph 2007	Hospitalizations per 12 months: 0	0
Hieftje et al	Bartholomew 2000	Hospitalization rates down 0.14 (Event rate – no units for this.)	n/a
	Rubin 1986	No difference in hospitalizations intervention vs control	0
	McPherson 2006	At one month no significant difference in hospital admissions	0
Johansen et al	Guendelman 2002	'there was no improvement in ….. number of hospital admissions,'	0
Kirk et al	Guendelman 2002	No significant differences between intervention group and control group for …….hospital admissions (OR 0.99; 95% CI 0.25, 3.88; *P* = 0.96).	0

**OUTCOME: ED VISITS**			
**Review**	**RCT**	**Results**	**Favors intervention group? [a]**
MacLean et al	Rasmussen 2005	One or more emergency department visits within 12 months:<br>Intervention group: events 2, participants 85<br>Control group: events 1, participants 168<br>Odds ratio: 4.02 (0.36 - 45.02) (M-H, Fixed 95% CI)	**0**
Stinson et al	Joseph 2007	ED visits per 12 months: +	**Y**
	Krishna 2003	Health service utilization (ED visits): +	**Y**
Hieftje et al	Bartholomew 2000	Effect size 0.03 - No difference in ED visits	0
Boyd et al	Homer 2000	Mean number in intervention group 0.86, mean in control group 0.73.	n/a
Johansen et al	Guendelman 2002	'there was no improvement in ….. occurrence of emergency room visits,' (pg 8)	0
Kirk et al	Guendelman 2002	No significant differences between intervention group and control group for A&E attendance or hospital admissions." (OR 0.59, 95% CI 0.26 to 1.35)	0
	Jan 2007	The effect of the interventions on use of Accident & Emergency Department attendance, and general practitioner/primary care consultations …..four studies reporting no effect. [including Jan 2007]	0

**OUTCOME: LUNG FUNCTION**			
**Review**	**RCT**	**Results**	**Favors intervention group? [a]**
MacLean et al	Rasmussen 2005	Odds ratios for improved FEV1 > 300ml over baseline after six months of intervention:<br>Internet versus specialist group: OR 3.26 (1.50 to 7.11), P = 0.002.<br>Internet versus GP group: OR 4.86 (1.97 to 11.94) , P < 0.001.	Y
	Van der Meer 2009	Change in mean pre-bronchodilator FEV1:<br>Internet group 0.24 litres<br>Usual care group -0.01 litres<br>"indicating an improvement in the telehealthcare arm's FEV1."	n/a
	Jan 2007	"at 12 weeks, children in both groups had significantly increased PEF. Between group differences are reported as non-significant."	0
Boyd et al	Shames 2004	FEV1 predicted %: -0.6 (3.0612) %(SE)<br>-0.60 [-6.60, 5.40] % (95% CI)<br>PEF litres per min: -21.4 (35.6378) L/min(SE)<br>-21.40 [-91.25, 48.45]	0
Johansen et al	Guendelman 2002	The intervention group was also significantly (P = .01) less likely to report peak flow readings	Y
	Rasmussen 2005	Treatment and monitoring with the Internet-based management tool led to more significant improvement in the Internet group than in the other 2 groups regarding asthma symptoms (Internet vs specialist: odds ratio2.64, P = .002; Internet vs GP: odds ratio 3.26; P < .001).<br>FEV1: number at randomisation in control (GP monitoring): 100, in intervention group1(internet monitoring) 100, in intervention group2	Y<br>(between internet vs GP group)

		(specialist monitoring): 100.<br>Within group pre-post change control = 0.004, intervention group1= 0.187, intervention group2 = 0.035<br>Between-group pre–post change between control and group 1: –0.183, P < .001.<br>Between-group pre–post change between control and group 2: –0.031 (Non-significant)	
Welsh et al	Kamps 2008	did not report any significant differences in lung function (FEF (forced expiratory flow) 25% to 75%) using repeated measures analyses of covariance and pooled time series analysis at two months (n = 10), six months (n = 6) and 12 months (n = 6).	0
Kirk et al	Jan 2007	No significant differences between groups for lung function.	0
	Guendelman 2002	Children in the intervention group…were less likely to report peak flow recordings in the yellow or red zone."<br>Intervention group reported fewer readings in red/yellow zones than control (OR 0.43; 95% CI 0.23, 0.82; *P* = 0.01).	Y
McDermott et al	Sundberg 2005	After intervention, FEV increased significantly in the intervention group compared to controls. (FEV Expt group change = 4.0, Cont = -0.55, p = 0.01). The improvement in FEV in the intervention group was significant (estimate 3.8%, p = 0.02) in a multiple linear regression model adjusting for age, sex, skin prick test, smoking habits and baseline FEV.	Y
	Huss A 1992	There were no significant differences in …. mean FEV.	0

**OUTCOME: QUALITY OF LIFE (QOL)**			
**Review**	**RCT**	**Results**	**Favors intervention group?** [a]
MacLean et al	Rasmussen 2005	Asthma Quality of Life Questionnaire (AQLQ):<br>"...data were not normally distributed and so median scores were supplied by author on request."<br>Internet group: AQLQ 6.42 (IQR 3.62 - 7.00)<br>GP group AQLQ: 6.31 (IQR 3.98 - 7.00)<br>Specialist group: AQLQ 6.17 (IQR 1.41 - 7.00)	n/a
	Van der Meer 2009	Mean AQLQ score:<br>Intervention group: 6.29 (SD 0.68), participants 91<br>Control group: 5.97 (SD 0.69), participants 92<br>Mean difference in AQLQ: 0.32 (0.12, 0.52) (IV, Fixed, 95% CI).	Y
	Jan 2007	Paediatric Caregiver's Quality of Life Questionnaire:<br>"...insufficient summary statistics were reported for meta-analysis. Only the caregivers of asthmatic children randomised to the intervention group showed significant improvement after the study compared to before the study."	Y
Stinson et al	Joseph 2007	QOL: 0	0
	Krishna 2003	QOL: 0	0
	Jan 2007	QOL: +	Y
Hieftje et al	Shames 2004	Intervention associated with higher scores in physical QOL measures at weeks 32 (score 78 vs. 59 p<0.05) and 52 (79.9 vs. 69.9 P<0.05)	Y
Johansen et al	Chan 2007	Caregivers in both groups perceived an increase in quality of life and an increase in asthma knowledge scores from baseline.	n/a
	Jan 2007	quality of life of caregivers were all significantly higher in the intervention group than in the conventional asthma care group.	Y
	Rasmussen 2005	Treatment and monitoring with the Internet-based management tool led to more significant improvement in the Internet group than in the other 2 groups regarding ….. quality of life (Internet	Y

		vs specialist: odds ratio 2.21, P = .03; Internet vs GP: odds ratio 2.10, P = .04)	
	Van der meer 2009	Asthma-related quality-of-life improvement of 0.5 point or more occurred in 54% and 27% of Internet and usual care patients, respectively (adjusted relative risk 2.00, confidence interval 1.38–3.04). Statistically significant, but not clinically significant.<br>Asthma related quality of life:<br>number at randomisation in control: 99, in intervention group: 101.<br>Within group pre-post change control = -0.18, intervention group = -0.56<br>Between-group pre–post change 0.38, P < .001.	Y
Welsh et al	Kamps 2008	Kamps 2008 measured physical function and psychosocial health with the generic PedsQL and asthma-related quality of life …. No significant treatment effects were observed using repeated measures analyses of covariance and pooled time series analysis using data from baseline (n = 15), two (n = 10), six (n = 6) and 12 months (n = 5)	0
Kirk et al	Jan 2007	Assessed via Pediatric Asthma Quality of Life Questionnaire. "Quality of life improved significantly in the intervention group (6.5 ± 0.5; *P* _ 0.05).	Y

**OUTCOME: SYMPTOMS**			
**Review**	**RCT**	**Extracted data**	**Favors intervention group?** [a]
MacLean et al	Guendelman 2002	Asthma control problems at 12 weeks:<br>between group difference: p= 0.07	0
	Rasmussen 2005	Symptom improvement over six months:<br>Internet versus specialist groups: OR: 2.64 (95% CI 1.43 to 4.88,P = 0.002);<br>Internet versus GP groups: OR: 3.26 (95%CI 1.71 to 6.19, P < 0.001).	Y
	Van der Meer 2009	ACQ change from baseline:<br>Internet group: - 0.54; Control group: - 0.06<br>adjusted difference - 0.47 (CI -0.64 to -0.3)<br>"slightly smaller than a clinically relevant difference of -0.5 (where negative change represents improvements)."	Y
	Jan 2007	Paediatric Asthma Control Test (ACT) change from baseline at 12 weeks: "intervention group had a significant decrease of night-time (P=0.028) and daytime (P = 0.009) symptoms compared to children in the control group. There were no between group comparisons for this study."	Y
Stinson et al	Jan 2007	symptom scores: +<br>asthma control: +	Y
	Joseph 2007	number of symptomatic days per two weeks: +<br>number of symptomatic nights per two weeks: +	Y
	Krishna 2003	decreased days with asthma symptoms: +	Y
Murray et al	Krishna 2003	Decrease in days with asthma symptoms at 12 months:<br>SMD: 0.40 (Lipsey Category: medium)	n/a
Hieftje et al	Bartholomew 2000	Intervention group lower symptom scores at post test, moderated by the severity of asthma (t(116) = -1.96; p=0.02)	Y
	Huss B 2003	No significant changes in symptom scores	0
	Shames 2004	No change in days with asthma symptoms at 8, 32, or 52 weeks (p=0.06). No difference in any of other clinical outcomes including wheezing days, asthma attacks.	0
Johansen et al	Chan 2007	There were no other differences in therapeutic or disease control outcome measures.	na
	Jan 2007	When the 2 groups were compared with regard to change from baseline, the children in the intervention group had a significant decrease of nighttime (P = .028) and daytime symptoms (P = .009) compared with	Y

		the children in the control group.	
	Rasmussen 2005	Treatment and monitoring with the Internet-based management tool led to more significant improvement in the Internet group than in the other 2 groups regarding asthma symptoms (Internet vs specialist: odds ratio2.64, P = .002; Internet vs GP: odds ratio 3.26; P < .001).	Y
	Van der Meer 2009	Asthma control improved more in the Internet group than in the usual care group (adjusted difference –0.47, confidence interval –0.64 to –0.30). At 12 months, 63% of Internet patients and 52% of usual care patients reported symptom-free days in the previous 2 weeks (adjusted absolute difference 10.9%, confidence interval 0.05%–21.3%).	Y
Welsh et al	Kamps 2008	measured caregiver-reported and child-reported asthma symptoms using PedsQL Asthma Module and found no significant difference in treatment effect at baseline and two, six and 12 months. Repeated-measures ANCOVAS did not yield significant interactions or main effects for symptoms cores.	0
Kirk et al	Jan 2007	Asthma symptoms significantly lower in intervention than control group" – (nocturnal *P* = 0.028; daytime *P* = 0.009).	Y
McDermott et al	Sundberg 2005	There was no significant effect of the intervention on prevalence of nocturnal and diurnal respiratory symptoms according to physicians' report, on self-report of prevalence of asthma symptoms or FVC.	0
	Huss A 1992	There were no significant differences in …. symptomatic improvement.	0

**OUTCOME: MARKERS OF SELF CARE**			
**Review**	**RCT**	**Extracted data**	**Favors intervention group? [a]**
Ring et al	Rasmussen 2005	"...significantly more patients using an internet-based AP reported using their AP at six months follow up (88%) than those who received an AP from a specialist via an out-patient clinic (66%) or those that received usual care from a GP (6%) (p<0.001) AP use was not defined beyond asking patients to say yes/no to whether they used their plan"	Y
Murray et al	Bartholomew 2000	Child self- management at 7.9 months (mean):<br>SMD: 0.32 (Lipsey Category: small)	n/a
		Child Self-efficacy expectations at 7.9 months (mean):<br>intervention group participants 70; control group participants 69<br>SMD: 0.14 (Lipsey Category small)	n/a
	Guendelman 2002	Percentage of patients taking asthma medication without reminder at 12 weeks: OR: 2.88 (>1 favours intervention)	n/a
	Homer 2000	Percentage of patients with peak flow metre available at greater than 9 months: OR: 0.91 (>1 favours intervention)	n/a
	Shegog 2001	Child self-efficacy for asthma self-management at 3 weeks<br>intervention group participants 38; control group participants 32<br>SMD: 0.48 (Lipsey Category medium)	n/a
Stinson et al	Jan 2007	"Diary adherence was significantly higher in internet group but declined over time in both groups"	Y
	Joseph 2007	"Internet students more likely to complete all four sessions compared to control group"	n/a
	Krishna 2003	"40–100% of parents completed three sessions; while 7- to 17-year olds who completed visit 3 had mastered 48–100% of material and completed 58% or more of the program"	n/a
Hieftje et al	Bartholomew 2000	Child Self- Management = 0.44	n/a
		Clinic appointment return rate was 71% (intervention) vs 60% (control) (P = 0.04)<br>….. found that experimental subjects, compared with the control group, had…… a significantly lower clinical appointment return rate (*P* = .04) [conflicting message so unable to interpret.	n/a
	Rubin 1986	Higher asthma behavioral child assessment score (mean +SD) in intervention (64.1+7.7) vs. control (57.8+8.8) (p=0.008)	Y
	Vilonzi 2001	Successful spirometry was achieved by 69.9% SpiroGame vs 47.1% of control (P = .002); FEV1 values achieved by 79% with SpiroGame but	Y

		4% with candle blowing (control group).	
Johansen et al	Chan 2007	(both studies). Virtual patients had higher metered-dose inhaler with valved holding chamber technique scores than did the office-based group at 52 weeks (94% vs 89%),had greater adherence to daily asthma symptom diary submission (35.4% vs 20.8%), had less participant time (636 vs 713 patient-months),	n/a
	Guendelman 2002	Self-care behaviors also improved far more for the intervention group.	n/a
	Jan 2007	The adherence rates of therapeutic and diagnostic monitoring, ….. were all significantly higher in the intervention group than in the conventional asthma care group.	Y
Kirk et al	Guendelman 2002	Self-care behaviours also improved more for the intervention group. However, it appeared that the 'Health Buddy' only brought about short term improvements as by 12 weeks both intervention and control groups had improved and no significant difference between them	0
McDermott et al	HussA 1992	According to participant self-report and objective assessments, the experimental group implemented significantly more allergen avoidance measures than controls at 12 weeks and also recorded a significant decline in mite-allergen levels in the home. The experimental group implemented significantly more avoidance measures (p = 0.023), showed a significant decline in mite-allergen levels (p = 0.004) There were no significant differences in allergen levels in living room carpets or sofas. [overall positive]	Y

**OUTCOME: KNOWLEDGE**			
**Review**	**RCT**	**Extracted data**	**Favors intervention group? [a]**
Stinson et al	Jan 2007	Asthma knowledge: +	Y
	Krishna 2003	Knowledge: +	Y
Murray et al	Bartholomew 2000	knowledge score at mean of 7.9 months:<br>SMD: 0.11 (Lipsey Category: small)	n/a
	Krishna 2003	Paediatric Asthma Knowledge Care Survey at 12 months:<br>SMD: 0.96 (Lipsey Category: large)	n/a
	Shegog 2001	Knowledge Questionnaire at 3 weeks:<br>SMD: 0.56 (Lipsey Category: large)	n/a
Johansen et al	Chan 2007	Caregivers in both groups perceived an increase in quality of life and an increase in asthma knowledge scores from baseline.	n/a
	Jan 2007	knowledge of asthma self-management, and quality of life of caregivers were all significantly higher in the intervention group than in the conventional asthma care group.	Y
Kirk et al	Jan 2007	Assessed via Asthma knowledge questionnaire (10 items) Asthma knowledge scores significantly higher in the intervention group than control group.(P ≤0.05)	Y

**OUTCOME: MEDICATION USE**			
**Review**	**RCT**	**Results**	**Favors intervention group? [a]**
Hieftje et al	McPherson 2006	Intervention group had lower use of oral steroids (OR 2.96, 95% CI 1.014-8.612, p=0.03)	Y
	Shames 2004	Days used rescue inhaler– no difference between control and intervention	0
Kirk et al	Jan 2007	"The intervention group reported ….. increased adherence….."	n/a

	Guendelman 2002	associated with a short –term (12 week) improvement in adherence to prescribed medications based on caregiver assessments	n/a
McDermott et al	HussA 1992	Decreased inhaled bronchodilator use per day (p = 0.023)	Y

a: Y = yes, 0 = no statistical difference; n/a = not applicable (unable to comment on statistical significance of provided results)
SMD: standardized mean difference
ACQ: Asthma Control Questionnaire
AQLQ: Asthma Quality of Life Questionnaire
OR: Odds ratio
